# Nonsense Suppression as an Approach to Treat Lysosomal Storage Diseases

**DOI:** 10.3390/diseases4040032

**Published:** 2016-10-19

**Authors:** Kim M. Keeling

**Affiliations:** Department of Biochemistry and Molecular Genetics, Gregory Fleming Cystic Fibrosis Research Center, Comprehensive Arthritis, Musculoskeletal, Bone, and Autoimmunity Center, University of Alabama at Birmingham, Birmingham, AL 35294, USA; kkeeling@uab.edu; Tel.: +1-205-975-6585

**Keywords:** lysosomal storage diseases, nonsense mutation, suppression, premature termination codon, readthrough, nonsense-mediated mRNA decay, translation termination, therapy, treatment

## Abstract

In-frame premature termination codons (PTCs) (also referred to as nonsense mutations) comprise ~10% of all disease-associated gene lesions. PTCs reduce gene expression in two ways. First, PTCs prematurely terminate translation of an mRNA, leading to the production of a truncated polypeptide that often lacks normal function and/or is unstable. Second, PTCs trigger degradation of an mRNA by activating nonsense-mediated mRNA decay (NMD), a cellular pathway that recognizes and degrades mRNAs containing a PTC. Thus, translation termination and NMD are putative therapeutic targets for the development of treatments for genetic diseases caused by PTCs. Over the past decade, significant progress has been made in the identification of compounds with the ability to suppress translation termination of PTCs (also referred to as readthrough). More recently, NMD inhibitors have also been explored as a way to enhance the efficiency of PTC suppression. Due to their relatively low threshold for correction, lysosomal storage diseases are a particularly relevant group of diseases to investigate the feasibility of nonsense suppression as a therapeutic approach. In this review, the current status of PTC suppression and NMD inhibition as potential treatments for lysosomal storage diseases will be discussed.

## 1. Premature Termination Codons Are Frequently the Cause of Disease

**Figure 1:**
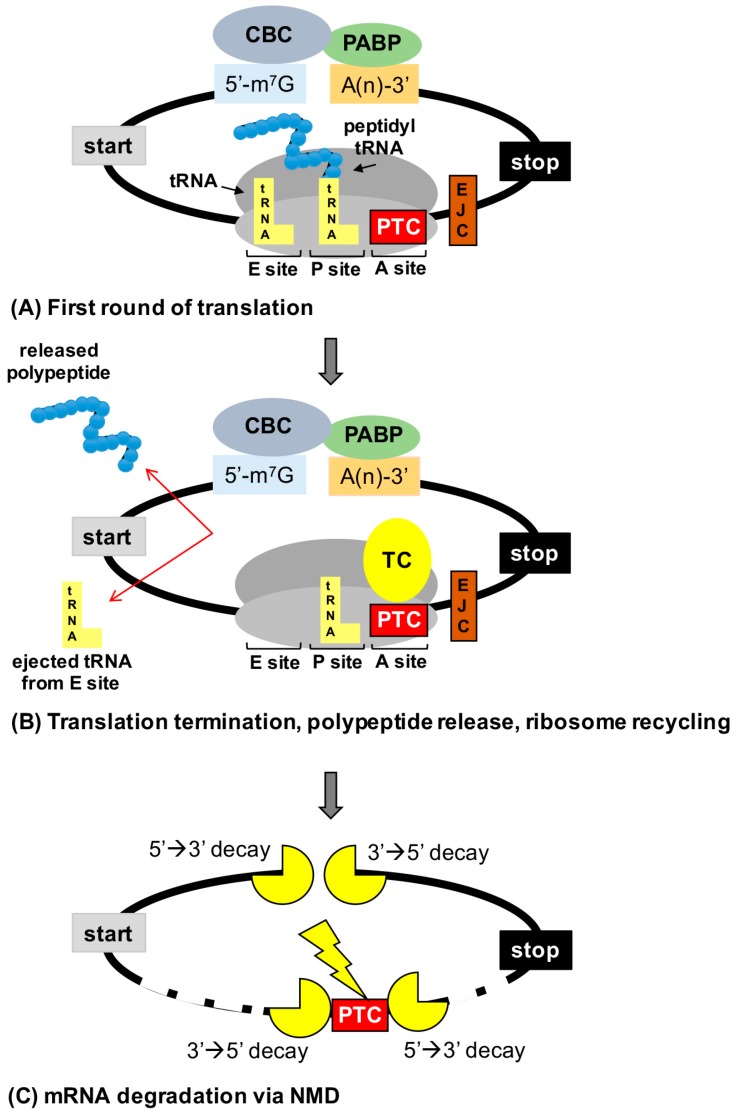
Consequences of premature termination codons (PTCs) on gene expression. (**A**) mRNAs are evaluated during the initial or “pioneer” round of translation for PTCs. mRNAs form a circular structure due to interactions between factors associated with the cap binding complex (CBC) and poly (A) binding protein (PABP). (**B**) Recognition of a PTC in the ribosomal acceptor (A) site by the termination complex (TC) leads to termination of translation before a full-length polypeptide is generated. (**C**) PTCs that lay 50–55 nucleotides upstream of an exon junction complex (EJC) are recognized by nonsense-mediated mRNA decay (NMD) factors that recruit the mRNA decay machinery to degrade the mRNA (either via the action of an endonuclease followed by degradation or by direct recruitment of decay factors to the 5′ or 3′ ends of the mRNA), preventing the mRNA from undergoing subsequent rounds of translation. A site = acceptor site, P site = peptidyl site, and E site = exit site.

Approximately one-third of all disease-associated gene lesions generate a premature termination codon (PTC) within the open reading frame of an mRNA [1]. A PTC reduces gene expression through two mechanisms (Figure 1). First, a PTC prompts translation elongation of an mRNA to terminate before a full-length polypeptide is generated, leading to the formation of a truncated polypeptide that lacks normal function and/or is unstable. Second, a PTC often elicits nonsense-mediated mRNA decay (NMD), a conserved cellular surveillance pathway that recognizes and degrades mRNAs harboring a PTC. Together, these two PTC mediated mechanisms can reduce the level of functional protein to such an extent that a disease phenotype develops. Because of the severe reduction in gene expression associated with PTCs, mutations that generate a PTC are generally associated with a more severe disease phenotype than missense mutations, for example, which may allow the retention of at least partial protein function.

In the past decade, significant progress has been made in the development of a novel therapeutic approach called nonsense suppression therapy, which targets translation termination at in-frame PTCs within mRNAs [2,3]. This therapeutic approach utilizes small molecular compounds that stimulate the insertion of an amino acid at the site of a PTC during mRNA translation. This mechanism suppresses translation termination at a PTC, allowing translation elongation of the mRNA to continue in the original ribosomal reading frame to generate a full-length polypeptide. This mechanism is also often referred to as “readthrough.” While PTCs can be formed by various types of gene lesions, it is single nucleotide substitutions that generate in-frame PTCs (also known as nonsense mutations) that are candidates for nonsense suppression since only suppression of in-frame PTCs has the potential of generating normal protein functionality. Significantly, nonsense mutations comprise 11% of all disease-associated gene lesions [4]. While the prevalence of nonsense mutations varies for different diseases and among different patient populations, it is likely that for most genetic diseases, at least a subset of patients will carry a nonsense mutation. According to the NIH Office of Rare Disease Research (http://rarediseases.info.nih.gov) and the National Organization for Rare Disorders (http://www.rarediseases.org), over 7000 distinct genetic diseases are known, affecting 10% of the human population. When considering that 11% of gene lesions results in a nonsense mutation, these data imply that ~3 million Americans and ~30 million people worldwide have diseases attributable to a nonsense mutation. Further development of nonsense suppression therapy offers the potential of a treatment for a subset of patients with a variety of genetic diseases that often have few, if any, available treatments.

## 2. Mechanism of PTC Suppression

### 2.1. Overview of Translation

The process of translating an mRNA into a protein occurs in four major steps: (1) initiation, when the ribosome assembles onto the mRNA at the correct start signal; (2) elongation, during which the ribosome generates a protein based on the sequence of mRNA codons; (3) termination, which occurs when a termination signal is recognized and the newly made protein is released from the ribosome; and (4) recycling of the ribosome and translation factors. During translation elongation [5], codons located in the ribosomal acceptor (A) site are decoded by eEF1A-bound tRNAs in a two-step process. First, interactions between the tRNA anticodon, the mRNA codon, and a portion of the ribosome referred to as the decoding center form of a series of hydrogen bonds that probe the geometry of codon-anticodon pairing. Based on this initial proofreading step, recognition of a tRNA that is not cognate with a codon results in its ejection from the A site, while recognition of a cognate tRNA is followed by GTP hydrolysis via eEF1A. This is then subsequently followed by a second kinetic proofreading step to further ensure that a cognate aminoacyl-tRNA is accommodated into the A site, followed by dissociation and recycling of eEF1A. Peptide bond formation then occurs between the amino acid carried by the accommodated A site tRNA and the adjacent peptidyl-tRNA (in the P site), resulting in the addition of the amino acid to the nascent polypeptide. With the assistance of eEF2, the ribosome then translocates to allow the next codon to enter the A site and the previous tRNA (in the ribosomal exit or E site) is removed. This process is repeated for each mRNA codon until a stop codon (UAA, UAG, UGA) enters the A site, signaling for translation termination to occur.

Stop codons are decoded by proteins called release factors rather than tRNAs. In eukaryotes, two release factors, eRF1 and eRF3, bind to form the canonical termination complex [6]. eRF1 recognizes stop codons (UAA, UAG, UGA) located in the ribosomal A site and catalyzes release of the nascent polypeptide. The three-dimensional structure of the eRF1 protein resembles the overall shape of a tRNA molecule [7], suggesting similar geometrical constraints among molecules that decode either sense or stop codons located in the ribosomal A site. eRF3 is a GTPase that binds to eRF1 and upon GTP hydrolysis, induces a conformational change in eRF1 that enhances stop codon recognition as well as polypeptide release [8,9,10,11,12]. While the process by which tRNAs decode sense codons has been well characterized, less is known of the mechanism behind stop codon decoding by the termination complex. Regions of amino acid sequence that lie in multiple locations within the N-terminal domain of eRF1 have been shown function to participate in stop codon recognition upon conformational changes in eRF1. In addition, evidence suggests that regions of the ribosome, including the decoding site, participate with eRF1 to decode stop codons. For example, genetic interactions have been found between the ribosomal decoding site and eRF1 in yeast [13]. Furthermore, recent cryo-electron microscopy studies of the mammalian termination complex indicated that the N-terminal domain of eRF1 contacts the ribosomal decoding site [8,14]. Thus, while decoding sense codons and stop codons require different machinery, many aspects of these decoding processes are similar.

Although eRF1 and eRF3 are considered to compose the canonical termination complex, several other factors have also been recently identified that modulate the activity of the termination complex. DDX19 (Dbp5 in yeast), a DEAD-box, ATP-dependent, RNA helicase that functions in the transport of mRNAs from the nucleus to the cytoplasm, has been shown to bind directly to eRF1 [15]. This interaction between eRF1 and DDX19 appears to aid in positioning eRF1 at stop codons and to promote complex formation between eRF1 and eRF3. DDX19 cofactors, GLE1 and IP6, appear to support the role of DDX19 in regulating eRF1 [16]. In addition, the factor ABCE1 (Rli1 in yeast) has been shown to interact with eRF1 in order to recycle ribosomes after peptide release [17,18]. Furthermore, the interaction between ABCE1 and eRF1 aids in stop codon recognition [19] and enhances the rate of peptide release [20]. Another factor, OGFOD1 (Tpa1 in yeast), has been shown to directly interact with both eRF1 and eRF3 and modulate stop codon recognition [21,22]. In addition, OGFOD1 functions as a prolyl hydroxylase that adds a hydroxyl group to a proline residue in the ribosomal protein RPS23 [23]. This post-translational modification of RPS23 alters the decoding of stop codons in a manner that is dependent on the mRNA context and on hypoxic conditions [24]. These studies suggest that multiple factors associate with the termination complex to influence stop codon decoding and that, in addition to the termination complex, these interacting factors may be pharmaceutical targets for the discovery of agents that promote PTC suppression.

### 2.2. Termination Suppression

**Figure 2:**
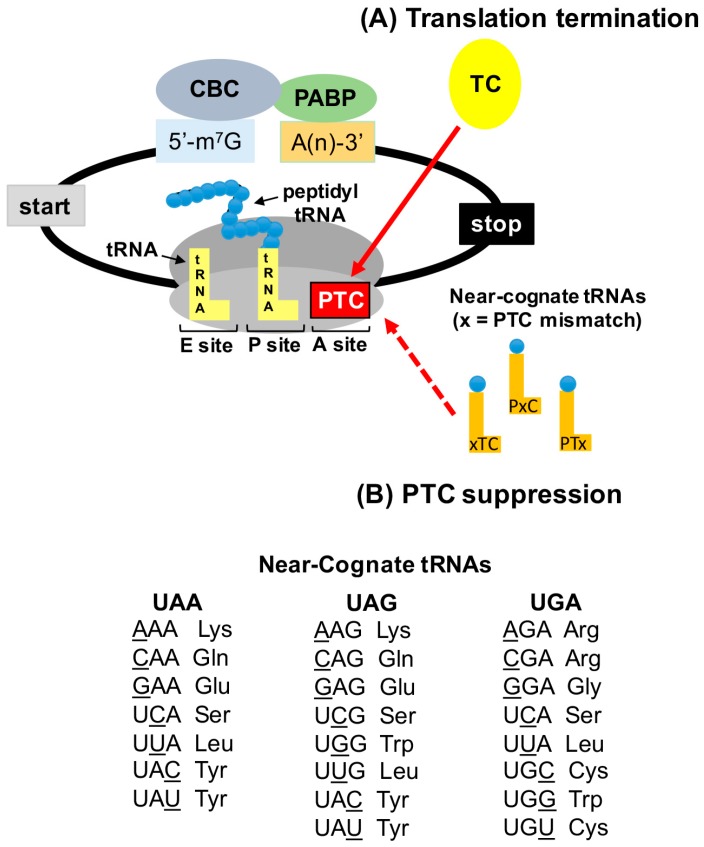
PTC suppression mechanism. (**A**) PTCs are recognized by the termination complex (TC), a process that is normally very efficient. (**B**) Near-cognate aminoacyl tRNAs (that base pair with two of the three nucleotides of the stop codon) can compete with eRF1 for PTC binding in the ribosomal A site. Accommodation of near-cognate aminoacyl tRNAs allows an amino acid to become incorporated into the nascent polypeptide at the site of the PTC, allowing translation to continue in the correct ribosomal reading frame to generate a full-length polypeptide. The “x” depicted in the schematic of near-cognate tRNAs indicates the nucleotide position of the PTC that is non-cognate to the near-cognate tRNA (xTC = first nucleotide, PxC = second nucleotide, and PTx = third nucleotide). The aminoacyl tRNAs that are near-cognate to stop codons are shown.

When a stop codon enters the ribosomal A site, a competition for accommodation occurs between eRF1 and aminoacyl-tRNAs. eRF1 vastly outcompetes tRNAs for stop codon binding because no tRNAs exist that are cognate to one of the three stop codons (UAA, UAG, or UGA). For example, normal stop codons at the end of open reading frames are generally suppressed at a rate of ≤0.1% [2]. However, the termination efficiency at PTCs is generally reduced, leading to an ~10-fold increase in the rate of PTC suppression relative to normal stop codons [2]. Ribosomal toe-printing experiments have indicated that termination at a PTC is slower than at a normal stop codon [25]. This difference in termination efficiency is likely due to additional interactions that occur between the termination complex and factors bound to the 3′ untranslated region (UTR). eIF4G, a component of the mRNA 5′ cap structure, associates with poly(A) binding protein (PABP) bound to the 3′ poly(A) tail to form a circular, closed loop messenger ribonucleoprotein (mRNP) structure [26] (Figure 1 and Figure 2). In addition, eRF3 also binds to PABP to enhance translation termination efficiency [27]. Because PTCs are spatially farther away from the 3′ UTR than normal stop codons, the interaction between the PTC-bound eRF3 and PABP will likely be less efficient than eRF3 bound to a normal stop codon. Thus, variations in termination complex binding partners at PTCs versus normal stop codons appear to contribute to differences in termination efficiency.

In addition to differences in the mRNP structure at a normal stop codon versus a PTC, it has also been shown that the identity of the stop codon, as well as the mRNA sequence flanking a stop codon, plays a significant role in termination efficiency and PTC suppression [28,29,30,31,32,33,34,35,36,37,38]. A number of studies suggest that, in general, UGA is the stop codon most susceptible to suppression, followed by UAG and UAA. This may be due to the series of hydrogen bonds that are formed between eRF1 and the different stop codons [14]. In addition, the mRNA sequences upstream and downstream of a stop codon [29,30,32,35,36,39], as well as the amino acids near the C-terminus of the nascent polypeptide [33], also affect the level of stop codon suppression. These studies have shown that in general, a cytosine following a stop codon increases the frequency of suppression. This is likely also related to the mechanism of stop codon recognition by eRF1, where it has been shown that in addition to the stop codon, eRF1 also contacts nucleotides downstream of a stop codon [14].

The proximal mRNA sequence surrounding stop codons that reside at the end of an open reading frame has been shown to often promote efficient translation termination, particularly among highly expressed transcripts [40]. This suggests an evolutionary conservation of efficient termination context at normal stop codons that protect cells against suppression of normal stop codons, which would generate proteins with C-terminal extensions that potentially acquire aberrant functions. However, the generation of C-terminally extended proteins due to stop codon suppression is also prevented by other cellular mechanisms. For example, nonstop mRNA decay is an mRNA degradation pathway that degrades transcripts with stalled ribosomes bound to the poly(A) tail due to either the absence of a stop codon, or the lack of stop codon recognition [41]. In addition, a protein degradation mechanism was recently identified that destabilizes proteins with a C-terminal extension resulting from suppression of natural stop codons. Sequences were found residing in the 3′ UTR that encode destabilizing peptides that protect cells against aberrant proteins produced from faulty termination [42]. Thus, several protective mechanisms exist that prevent normal stop codon suppression or eliminate gene products resulting from normal stop codon suppression. These protective measures also suggest that nonsense suppression therapy is unlikely to induce normal stop codon suppression that would cause the onset of aberrant phenotypes.

It has also recently been elucidated that mammalian cells utilize stop codon suppression (also termed readthrough) as a means of expanding and/or controlling gene expression. For example, the vascular endothelial growth factor A (*VEGFA*) mRNA in mammalian endothelial cells undergoes programmed stop codon readthrough to generate VEGF-Ax, a unique protein isoform with a C-terminal extension of 22 amino acids [43]. VEGF-Ax exhibits antiangiogenic activity in contrast to the proangiogenic function of VEGFA. A *cis* element in the 3′ UTR of *VEGFA* that is recognized by the *trans* acting factor A2/B1 heterogeneous nuclear ribonucleoprotein (hnRNP) promotes decoding of the UGA stop codon as a serine. Significantly, VEGF-Ax expression has shown to be depleted in colon adenocarcinomas. Two other mammalian transcripts, *MTCH2* and *AGO1* were also identified to carry a similar 3′ UTR cis-acting element that elicits stop codon suppression [43]. Additional mammalian transcripts that have been identified as producing proteins with an extended C-terminus due to stop codon readthrough include rabbit beta-globin [44], rat myelin protein zero, which may play a role in myelination [45], and peroxisomal lactate dehydrogenase, which likely functions in redox equivalent regeneration in peroxisomes [46]. Furthermore, the efficiency of translation termination can be altered in response to a variety of stress stimuli through hydroxylation of various ribosomal proteins, as well as translation elongation and termination factors [47]. Thus, the efficiency of termination at stop codons represents a way that cells regulate gene expression and expand the proteomic repertoire.

While the exact mechanism underlying PTC suppression has yet to be elucidated, studies have identified the amino acids that become incorporated during stop codon suppression in yeast. In these studies, reporter proteins generated from suppression of a PTC were purified and subjected to either Edman sequencing [48] or to tandem mass spectrometry [49,50] in order to identify the amino acids that become incorporated during PTC suppression. The amino acid most frequently incorporated during UGA readthrough was tryptophan, followed by cysteine and arginine [49,50]. Blanchet et al. found that tyrosine was the amino acid most frequently incorporated at UAA and UAG, followed by glutamine and lysine [49]. Alternatively, Roy et al. found that glutamine was the most frequently incorporated amino acid at UAA and UAG, followed by tyrosine and lysine [50]. Fearon et al. identified tyrosine, lysine, and tryptophan as the amino acids incorporated at UAG [48]. These results suggest that amino acids are not randomly inserted at PTCs during suppression. Rather, these data suggest that the amino acids inserted at PTCs during suppression are carried by near-cognate tRNAs, which can base pair with two of the three nucleotides of a stop codon. However, as indicated in Figure 2, which depicts the aminoacyl tRNAs that are near-cognate to stop codons, not all of the amino acids that are carried by near-cognate are incorporated during PTC suppression. These observations provide important considerations for the effectiveness of nonsense suppression therapy to restore deficient protein function. For example, these data indicate that an amino acid may not necessarily be inserted at a PTC that restores wild-type protein functionality. Thus, a variant protein may be produced by PTC suppression that possesses only partial protein function. This must be taken into consideration when examining PTC suppression as a therapeutic strategy in terms of the total amount of protein function that is required to alleviate a disease phenotype, as well as the possibility that for proteins consisting of multimers, the generation of a variant protein could potentially act in a dominant negative manner. Interestingly, these yeast studies also indicate that, although the subset of amino acids inserted during PTC suppression remains the same, the proportions of these amino acids change when different conditions are used to stimulate suppression [48,49,50]. This further suggests that different classes of nonsense suppression compounds may influence the proportions (or possibly the identities) of amino acids that become incorporated at PTCs, and that nonsense suppression compounds need to be selected for the ability to incorporate amino acids that generate the highest level of protein function. This insight is also relevant to drug discovery strategies to discover new, more effective nonsense suppression compounds. Many reporter assays evaluate PTC suppression efficiency based solely on the general ability of a compound to insert an amino acid at a PTC rather than the ability of a drug to incorporate an amino acid that restores protein function. Thus, one nonsense suppression agent might be more effective for a particular mutation than another based on multiple factors, including the identity of the PTC, the sequence surrounding the PTC, and the proportions of amino acids inserted during readthrough. All of these factors must be considered for the selection of the most efficient nonsense suppression compound for a particular disease-associated nonsense mutation.

## 3. Nonsense Suppression as a Treatment for Lysosomal Storage Diseases

In 1996, the first study that examined PTC suppression as a therapeutic approach to restore deficient protein in the context of a genetic disease was published [51]. Since that initial study, nonsense suppression therapy has been explored as a potential therapeutic approach in more than 100 different publications for approximately 50 different genetic disorders [2,3,52]. The main limiting factor for nonsense suppression therapy is producing enough functional protein to alleviate a disease phenotype, which varies among different genetic diseases depending on the location, function, and expression level of the deficient protein.

Lysosomal storage diseases (LSDs) are a group of nearly 60 inherited metabolic disorders involving errors in lysosomal catabolic function [53]. While each of these disorders alone is rare, collectively, LSDs occur in approximately 1 in 8,000 live births. Catabolism and recycling of multiple substrates, including glycosaminoglycans, sphingolipids, glycogen, and proteins occur in the lysosome. Approximately 60 different hydrolases participate in the various lysosomal catabolic pathways. Loss of proper function, activation, or targeting of one of these lysosomal proteins prevents the degradation of its substrate, leading to accumulation of the substrate within lysosomes and onset of a specific LSD. Various LSDs exhibit different clinical manifestations depending on the substrate impacted and its site and level of accumulation. Different LSDs can manifest abnormalities within visceral, ocular, hematological, skeletal, and neurological tissues. The quality of life and life expectancy of LSD patients is often severely impacted. Importantly, neurological abnormalities are present in around two-thirds of LSD patients. Treating the ocular, skeletal, and neurological clinical manifestations associated with LSDs is challenging because these tissues are inaccessible to current treatment approaches. New treatments are sorely needed to address the clinical manifestations that are recalcitrant to current therapeutic options. Nonsense suppression therapy may be a viable treatment for LSD patients that harbor nonsense mutations. While the frequency of nonsense mutations varies among different LSD patient populations, nonsense mutations are particularly prevalent among some LSD patient populations. For example, it has been estimated 50%–70% of patients with the LSD Mucopolysaccharidosis I-Hurler (MPS I-H) carry a nonsense mutation [2].

LSDs are a group of disorders that are particularly relevant for suppression therapy due to their relative low threshold for correction compared to many other genetic diseases. For example, the amount of protein function that needs to be restored in order to alleviate cystic fibrosis [54] or Duchenne muscular dystrophy [55], two diseases that have rigorously been investigated as candidates for nonsense suppression therapy, has been estimated to be around 25%–35% of wild-type levels. However, for Mucopolysaccharidosis I-Hurler (MPS I-H), an LSD that is caused by the loss of the enzyme α-L-iduronidase, less than 1% of normal protein activity can significantly alleviate the disease phenotype [56].

Seventeen different nonsense suppression studies have been published to date that have examined the potential of using nonsense suppression as a potential treatment for various LSDs, including: Mucopolysaccharidosis I (MPS I), Mucopolysaccharidosis III (MPS III), Mucopolysaccharidosis VI (MPS VI), cystinosis, neuronal ceroid lipofuscinosis, and Niemann-Pick disease. Table 1 summarizes the LSD nonsense suppression studies that have been performed to date. In all of these LSD studies, improvements were observed in at least some of the experimental endpoints examined due to restoration of partial protein function. These data suggest that nonsense suppression therapy has the potential to be developed into a viable treatment option for LSD patients that harbor a nonsense mutation. The discovery and development of safe and effective nonsense suppression drugs is key to implementing nonsense suppression as a therapeutic approach.

### 3.1. Aminoglycosides

A number of small molecular weight compounds have been identified that suppress PTCs in mammalian cells. The best characterized of these compounds are the aminoglycosides, a class of structurally related antibiotics composed of a 2-deoxystreptamine ring linked to one or more amino sugars. Aminoglycosides bind to the ribosomal decoding site [74,75,76], a region of the ribosome that monitors the geometry of interactions within the ribosomal A site, including codon/anticodon interactions between the mRNA and aminoacyl tRNAs, as well as interactions between stop codons and eRF1. In bacteria, aminoglycosides bind tightly to the decoding site and at low doses, lead to translational misreading at sense and stop codons [2,77]. At high doses, aminoglycosides inhibit translation in bacteria, which serves as the mode of action for aminoglycosides as antibiotics. Because aminoglycosides bind much less efficiently to the eukaryotic decoding site, translational misreading in eukaryotes is largely circumvented [78,79], allowing these drugs to be safely administered as antibiotics. However, aminoglycosides have been shown to induce misreading at very low levels in mammalian cells [80,81,82], with the primary effect being increased suppression at PTCs. A subset of aminoglycosides has been shown to be effective at suppressing termination at PTCs both in cultured cells and animal models [3]. Several small clinical trials carried out with the aminoglycoside gentamicin found limited phenotypic improvements in a subset of cystic fibrosis [83,84] and Duchenne muscular dystrophy [85,86,87,88] patients who harbor nonsense mutations. In addition, aminoglycosides have been shown to suppress termination of nonsense mutations associated with several LSDs in cultured cells as well as in mouse models as indicated in Table 1.

Molecules that enhance PTC suppression by aminoglycosides have also been identified. A recent high throughput screen identified five compounds (CDX3, CDX4, CDX5, CDX10, CDX11) that potentiate PTC suppression by aminoglycosides [89]. CDX5, the most potent among the compounds identified, was able to increase PTC suppression in vitro by G418 up to 180-fold compared to treatment with G418 alone. The polyanion poly-L-aspartic acid (PAA) has also been shown to enhance aminoglycoside-mediated PTC suppression. PAA was previously shown to weaken interactions between aminoglycosides and membrane phospholipids within lysosomes [90,91]. This resulted in increased cytoplasmic aminoglycoside concentrations, as well as reduced aminoglycoside toxicity. A recent study indicated that relative to reporter cells treated with gentamicin alone, co-administration of PAA with gentamicin increased the level of PTC suppression by 20%–40% [92]. Similarly, co-administration of PAA with gentamicin in a nonsense mouse model of cystic fibrosis resulted in the restoration of more CFTR function than mice treated with gentamicin alone [92].

However, the use of traditional aminoglycosides for chronic, long-term nonsense suppression therapy is not feasible because aminoglycosides have the potential to induce ototoxicity [93,94,95,96,97] and nephrotoxicity [98,99,100]. The toxicity associated with aminoglycosides is largely due to mechanisms that are unrelated to the interactions of aminoglycosides with cytoplasmic ribosomes. This includes the ability of aminoglycosides to alter mitochondrial ribosome function [94,101,102] and the interaction of aminoglycosides with membrane phospholipids, which can inhibit phospholipase function [103,104,105,106,107]. To address the limitations of traditional aminoglycosides for nonsense suppression therapy, a medicinal chemistry approach has been pursued to design new aminoglycosides with an increased ability to suppress PTCs, while also being less toxic. This was accomplished by designing aminoglycosides that bind more efficiently to cytoplasmic ribosomes in order to induce higher levels of PTC suppression, while also reducing the affinity of the aminoglycosides for mitochondrial ribosomes, which plays a significant role in aminoglycoside toxicity [66,108,109,110]. Several generations of these designer aminoglycosides have been tested in numerous disease models, including cystic fibrosis [111,112], MPS I-H [67,69,70], Usher syndrome [113,114,115], and Rett syndrome [116,117]. These studies demonstrate that the rationale to create new aminoglycosides with reduced toxicity and enhanced PTC readthrough by enhancing their affinity for the cytoplasmic ribosome is valid.

One of these designer aminoglycosides, NB84, was recently investigated in a mouse model of MPS I-H that carries a PTC homologous to the *IDUA-W402X* nonsense mutation, the most common mutation among the MPS I-H patient population. This *Idua-W402X* mouse recapitulates many of the biochemical, morphological, and functional abnormalities found in MPS I-H patients [67,70,118]. Treatment with NB84 for 28 weeks was able to attenuate progression of the MPS I-H phenotype in the heart, bone, and brain of treated *Idua-W402X* mice compared to untreated controls [70]. This study showed, for the first time, that nonsense suppression therapy could moderate the progression of an LSD in vivo. Furthermore, this study indicated that nonsense suppression may be able to alleviate MPS I-H phenotypes in tissues such as the brain, bone and heart valves, which are inaccessible to current MPS I-H therapies such as hematopoietic stem cell transplantation (HSCT) [119] and enzyme replacement therapy (ERT) [120]. In agreement with other MPS I-H studies [121,122,123], early treatment intervention with nonsense suppression therapy in the *Idua-W402X* mouse model produced the most robust therapeutic results. While no evidence of toxicity was found in mice after long-term NB84 treatment, more extensive safety and toxicity studies are needed to determine whether NB84 and other new designer aminoglycosides are safe for long-term human use. The development of safer, more effective aminoglycosides for nonsense suppression therapy marks an important step forward in the development of more efficacious nonsense suppression drugs for treating LSDs, as well as other genetic diseases caused by nonsense mutations.

### 3.2. PTC124

Efforts have also been made to identify PTC suppression agents from screening libraries of small molecular weight compounds. High throughput screens of 800,000 low molecular weight compounds identified a novel readthrough compound called PTC124 (Ataluren, Translarna^TM^) [124]. The chemical structure of PTC124 is unrelated to that of aminoglycosides. Also unlike aminoglycosides, PTC124 does not have antibacterial properties and it is orally bioavailable. Furthermore, PTC124 has been found to be nontoxic and safe for human use [125,126]. Numerous cell-based studies have shown that PTC124 can suppress PTCs and restore function of deficient proteins [127]. Among LSDs, PTC124 has been shown to restore enzyme activity in cultured fibroblasts and lymphoblasts derived from patients with infantile neuronal ceroid lipofuscinosis [64] and in cultured fibroblasts derived from MPS VI patients [68] (Table 1). Short-term, two-day administration of PTC124 to *Cln1-R151X* mice, a model of nonsense infantile neuronal ceroid lipofuscinosis, revealed an increase in palmitoyl-protein thioesterase activity in the liver and muscle of PTC124-treated mice relative to controls [71]. However, no increase in enzyme activity was observed in other tissues. PTC124 has also been reported to improve biochemical endpoints in multiple tissues, including the brain, heart, spleen, liver, and lungs of an *Idua-W402X* mouse model of MPS I-H after two weeks of treatment [127,128].

Promising results from studies that evaluated the ability of PTC124 to restore deficient protein function in Duchenne muscular dystrophy (DMD) [124] and cystic fibrosis (CF) [129] nonsense mouse models led to the initiation of clinical trials in DMD and CF patients who harbor nonsense mutations. Proof of concept Phase 2a studies for DMD [130] and CF [131] showed that PTC124 (ataluren) treatment restored partial levels of full-length dystrophin and CFTR proteins, respectively. Based on these promising results, large-scale, randomized, double-blinded, placebo-controlled, international 48-week clinical trials were initiated for both DMD and CF. A previous observational study indicated that the 6-minute walk distance (6MWD) test, which measures the distance that a patient walks within a 6-minute period, is a useful endpoint to monitor clinical improvements in DMD patients [132]. The 6MWD test and quantitative strength assessments were used to evaluate the effectiveness of PTC124 to alleviate DMD in patients who harbor nonsense mutations [133]. After 48 weeks of PTC124 treatment, it was found that PTC124 was well-tolerated and a trend toward improvements in the 6MWD test and muscle strength endpoints were observed compared to the placebo cohort that suggested PTC124 slowed DMD progression. These results led the European Medicines Agency to grant a conditional marketing authorization of PTC124 (Translarna^TM^) as a treatment for DMD in ambulatory patients that are at least 5 years of age and carry a nonsense mutation. Ongoing confirmatory studies are underway to verify the ability of PTC124 to alleviate DMD. The PTC124 clinical trials among CF patients showed no significant difference between lung function endpoints in PTC124-treated patients and those administered placebo [131]. However, stratification of the data based on tobramycin usage, which was subsequently found to inhibit PTC124 readthrough activity, indicated that patients treated with PTC124 who were not receiving inhaled tobramycin, showed improvement in lung function [131]. Additional PTC124 Phase 3 clinical trials are underway in CF patients not receiving chronic tobramycin therapy. In addition, proof-of-concept trials are underway in patients with the eye disorder aniridia (ClinicalTrials.gov identifier NCT02647359). Furthermore, PTC124 (Translarna^TM^) has been granted orphan drug designation in the U.S. and Europe for the treatment of the LSD MPS I-H (http://ir.ptcbio.com/releasedetail.cfm?releaseid= 888466) and proof-of-concept clinical studies for MPS I-H are underway (EU clinical trials register number 2014-002596-28). The variety of diseases that are currently being evaluated in PTC124 clinical trials suggests that if effective, PTC124 may be a drug that is generally applicable to many diseases caused by nonsense mutations, including LSDs. Because less protein function is required to alleviate many of the defects associated with LSDs compared to many genetic disorders, PTC124 may prove to be an effective treatment for LSDs.

### 3.3. Other Readthrough Compounds

Readthrough compounds other than PTC124 have also been identified from screening libraries of small molecular weight compounds. A screen of 34,000 compounds using an ELISA-based assay to find drugs that suppressed nonsense mutations in the *ATM* gene that are associated with ataxia telangiectasia (AT) identified several non-aminoglycoside compounds with readthrough activity [134]. Two of the most effective compounds, RT13 and RT14, were found to restore ATM kinase function in AT patient fibroblasts [134] and to restore full-length dystrophin protein in myotubes derived from a nonsense mouse model of DMD [135]. Derivatives of these compounds were also identified that suppress *ATM* nonsense mutations in AT lymphoblasts [136]. RTC13 and RTC14, as well as the derivatives BZ6 and BZ16, were also tested among several cultured LSD fibroblast cells lines, but no significant increases in enzyme activity were observed [72]. Other compounds that promote PTC suppression have also been identified and include a subset of macrolide antibiotics [137,138], as well as the peptide antibiotic negamycin [139,140,141,142]. In addition, the anti-inflammatory amlexanox was also shown to suppress multiple disease-associated nonsense mutations in mammalian cells [143]. Furthermore, derivatives of PTC124 were recently generated in which the fluoroaryl moiety was altered. These nontoxic PTC124 derivatives were found to be more effective than PTC124 at suppressing a UGA nonsense mutation in reporter cells as well as in a bronchial epithelial cell line derived from a cystic fibrosis patient. These data suggest that the structure of PTC124 may be further optimized to mediate more efficient suppression at some PTCs [144]. Furthermore, PTC-414, another novel nonsense suppression drug identified by PTC Therapeutics, was found, along with PTC124, to suppress a nonsense mutation in a zebrafish model for choroideremia, a chorioretinal dystrophy. Both PTC124 and PTC-414 restored enough protein function to increase embryo survival and prevented the onset of retinal degeneration [145]. These data suggest that multiple chemical scaffolds may be available for the development of nonsense suppression drugs and that additional, undiscovered chemical scaffolds may be available for the development of new, potent nonsense suppression drugs.

### 3.4. Inhibition of Nonsense-Mediated mRNA Decay (NMD)

In addition to producing a truncated polypeptide, another consequence of a PTC residing within the open reading frame of an mRNA is a decrease in the steady state mRNA levels through activation of nonsense-mediated mRNA decay (NMD). NMD is a conserved eukaryotic cellular surveillance pathway that recognizes and degrades mRNAs that harbor a PTC [146,147,148,149]. As mRNAs undergo splicing in the nucleus, a subset of NMD factors associate with transcripts as part of the exon-junction complex (EJC) that is deposited onto an mRNA 20–24 nucleotides upstream of each exon-exon junction. In the initial or “pioneer” round of translation, the ribosome displaces EJCs from an mRNA, which serves to remodel the mRNA for subsequent rounds of steady state translation. However, if a ribosome encounters a PTC that is at least 50–55 nucleotides upstream of an EJC, mRNA decay factors are recruited that degrade the mRNA. By reducing the abundance of PTC-containing mRNAs, NMD is thought to act as a protective mechanism for the cell by reducing the expression of truncated proteins that could potentially have deleterious, dominant-negative functions. However, by reducing the pool of mRNAs available for translation, NMD negatively impacts the therapeutic suppression of PTCs. Thus, inhibition of NMD may enhance the effectiveness of nonsense suppression drugs to restore protein function by increasing steady state mRNA abundance.

The efficiency of NMD can have important effects on disease phenotypes. Variability in NMD efficiency among patients that carry PTCs alters the inheritance pattern and the clinical severity of numerous diseases [150,151]. For some disorders, NMD prevents the expression of a protein that has dominant negative effects and can adversely affect the disease phenotype. For other disorders, NMD prevents the expression of a truncated protein with partial function that if expressed, could attenuate the disease phenotype. By inhibiting NMD, the steady state levels of the functional truncated protein can be increased. For example, inhibition of NMD has been shown to increase truncated 4.1R protein levels in hereditary elliptocytosis lymphoid cells [152] and the level of truncated collagen VI protein in Ullrich′s disease fibroblasts [153,154], which resulted in attenuation of these diseases in vitro. For other disorders caused by PTCs, the combination of nonsense suppression with NMD inhibition can restore full-length protein function to a greater extent than nonsense suppression alone. For example, in immortalized bronchial epithelia derived from cystic fibrosis patients who carry *CFTR* nonsense mutations, combining readthrough with NMD inhibition restored more CFTR function than either treatment strategy alone [155]. Similarly, in mouse embryonic fibroblasts derived from homozygous *Idua-W402X* mice, a model of the MPS I-H LSD, co-treatment with aminoglycosides in combination with the NMD inhibitor compound NMDI-1 restored more enzyme function than treatment with aminoglycosides alone [69]. In vivo co-treatment of aminoglycosides with NMDI-1 in *Idua-W402X* mice also improved biochemical endpoints to a greater extent than treatment with aminoglycosides alone [69]. These findings suggest that NMD attenuation may be a feasible approach to restore expression of functional truncated proteins, or in conjunction with a PTC suppression compound, to restore expression of functional full-length proteins. In support of NMD attenuation being used as a possible therapeutic approach, a number of compounds have been identified that attenuate NMD [156], including amlexanox, which appears to have a dual function as a readthrough drug and NMD inhibitor [143]. This suggests that additional compounds may be discovered that have dual readthrough/NMD inhibition functions that could be more effective at restoring deficient protein function than drugs that mediate either function alone. The search for dual-purpose compounds may be one approach to identify more effective nonsense suppression drugs.

While preliminary proof-of-principal studies indicate that NMD inhibition shows promise as a way to target PTC-mediated reductions in gene expression, caution must be employed with this approach. In addition to regulating the abundance of PTC-containing mRNAs generated by genomic mutations, NMD also affects the abundance of ~10% of the mammalian transcriptome [157]. Furthermore, NMD function is essential for mammalian embryonic development [158]. NMD and/or NMD factors are also known to function in diverse cellular pathways, including DNA synthesis, cell-cycle progression, telomere length homeostasis, and cellular stress responses [159]. NMD factor insufficiency is also associated with multiple forms of intellectual disability [160,161]. However, it has further been shown that NMD efficiency can vary by as much as 4-fold among the general population [155,162,163,164], suggesting that a certain degree of variability in NMD efficiency can be safely tolerated. Studies are underway to characterize the physiological role of NMD inhibition after the completion of development in order to determine whether attenuation of NMD is a safe, long-term therapeutic approach for diseases resulting from PTCs.

## 4. Consideration of Personalized Medicine Approaches for LSDs

Traditionally, a broad approach has been applied to the development of treatments for genetic diseases in an attempt to benefit the greatest number of patients possible. For example, significant success has been achieved with developing enzyme replacement therapies (ERT) for LSDs [165]. These treatments have led to significant improvements in the disease phenotypes and have increased the lifespan of patients. However, some tissues, such as the bone, brain, heart valves, and cornea are inaccessible to these treatments, and are therefore, recalcitrant. The clinical phenotypes associated with these tissues are often crucial to the quality of life for LSD patients. While nonsense suppression and NMD attenuation are personalized therapy approaches that target only a genotypic subset of patients that harbor an in-frame PTC, evidence suggests that the small molecular weight compounds that suppress PTCs and/or inhibit NMD can access these normally protected tissues to some extent [69,70,128], and may offer a substantial improvement in the quality of life for a subset of LSD patients. In addition, a personalized medicine strategy has recently been successful in the development of treatments for cystic fibrosis. The Cystic Fibrosis Foundation, which drives much of the research related to cystic fibrosis (CF), has aggressively supported not only CF clinical research, but basic research as well. This approach has led to a greater understanding of CFTR function in patients that carry different types of mutations, which have different effects on CFTR expression, localization, and function. Subsequently, drugs have been identified that target the specific defects associated with different classes of mutations [166,167,168]. These efforts have led, for the first time, approval of several drugs that specifically treat the CFTR defect in CF patients. This breakthrough in therapeutic strategy and drug discovery can be applied to other diseases as well, including LSDs. A combination strategy is another approach that has been implemented to treat CF patients that combines drugs that hit multiple targets (such as enhancing CFTR processing and increasing CFTR channel activity), which has led to treatment of the common ΔF508 mutation [168]. It is likely that this combination approach may also be required to treat LSDs, particularly in tissues such as the brain and the bone, which are resistant to current therapies. In order to restore a therapeutic level of protein function among recalcitrant tissues, nonsense suppression therapy and/or NMD inhibition may be viable options to complement other LSD treatment options, including: enzyme replacement therapy, the exogenous administration of purified recombinant enzyme [165]; substrate reduction therapy, which utilizes molecules that reduce the synthesis of the accumulation substrate [169]; and chaperone therapy, which uses compounds to stabilize misfolded, but functional, proteins [170]. With the continued development of more effective drugs, nonsense suppression therapy, alone or in combination with other therapeutic approaches, is likely to be a future treatment option for LSDs, as well as other genetic disorders, in patients who harbor nonsense mutations.

## Figures and Tables

**Table 1:** Nonsense Suppression Studies in lysosomal storage diseases (LSD) Models.

Year	LSD	Gene	Model	Mutation	Drug	Major Findings	Reference
2001	MPS I-H	*IDUA*	-Cell-free translation system -Cultured patient fibroblasts	-Q70X (UAG) -W402X (UAG)	-Gentamicin	-Q70X more susceptible to RT than W402X in cell free system -3% of normal enzyme activity restored in fibroblasts -GAG storage normalized -Lysosomal proliferation normalized	Keeling et al. [57]
2001	Infantile neuronal ceroid lipofuscinosis	*TPP1 or CLN2*	-Cultured patient fibroblasts	-Q66X (UAG) -R127X (UGA) -R208X (UGA)	-Gentamicin	-7% of normal enzyme activity restored for R127X allele -0.5% restored for R208X allele -None restored at Q66X allele	Sleat et al. [58]
2002	Cystinosis	*CTNS*	-Cultured patient fibroblasts -Reporter in HEK293 cells	W138X (UGA)	-Gentamicin	-Cysteine levels significantly reduced compared to controls -Detection of reporter protein in the presence of drug	Helip-Wooley et al. [59]
2002	MPS I-H	*IDUA*	-Cell-free translation system	W402X (UAG)	-Amikacin -Gentamicin -Tobramycin	-Tobramycin least effective -W402X context more resistant to RT than other UAG contexts	Keeling et al. [60]
2004	MPS I-H	*IDUA*	-Cultured patient fibroblasts -cDNAs in CHO-K1 cells	-Q70X (UAG) -W180X (UGA) -Y343X (UAG) -Q400X (UAG) -W402X (UAG) -R628X (UGA)	-Gentamicin	-Significant increases in enzyme activity observed in all treated fibroblasts except Y343X -All CHO-K1 cell lines responded with an increase in enzyme activity (UGA>UAG>UAA)	Hein et al. [61]
2009	MPS I-H	*IDUA*	-Cell-free translation system	-Q70X (UAG) -W402X (UGA)	-Gentamicin -NB30 -NB54 -Paramomycin	-NB30 & NB54 more effective at suppressing PTCs than paramomycin & gentamicin -New drugs shown to be less toxic than traditional aminoglycosides	Nudelman et al. [62]
2010	MPS I-H	*IDUA*	-Cell-free translation system	Q70X (UAG)	-Gentamicin -NB30 -NB54 -NB74 -NB84	-Novel aminoglycoside derivative NB84 is most effective at suppressing PTC -NB84 also less toxic than traditional aminoglycosides	Nudelman et al. [63]
2011	Infantile neuronal ceroid lipofuscinosis	*PPT1 or CLN1*	-Cultured patient fibroblasts -Cultured patient lymphoblasts	-L10X (UAG) -R151X (UGA) -R164X (UGA) -Q291X (UAG)	-Gentamicin -PTC124	-Both drugs restore enzyme activity in fibroblasts (~1%) and lymphoblasts (~0.3%) -Cell toxicity observed with gentamicin, but not PTC124 -PTC124 treatment restored full-length PPT1 protein, decreased ceroid levels & granular deposits, suppressed apoptosis	Sarkar et al. [64]
2011	MPS I-H	*IDUA*	-Cell-free translation system -Reporter in HEK293 cells	Q70X (UAG)	-Gentamicin -NB30 -NB54 -Other NB derivatives	-New aminoglycoside derivatives more effective than gentamicin at suppressing Q70X -Derivatives show less cell toxicity than gentamicin	Kandasamy et al. [65]
2012	MPS I-H	*IDUA*	-Cell-free translation system	Q70X (UAG)	-G418 -Gentamicin -NB124 -Other NB derivatives	-New synthetic aminoglycosides suppressed the Q70X mutation much more effectively than gentamicin or G418 -Derivatives showed less cell toxicity than traditional aminoglycosides	Kandasamy et al. [66]
2012	MPS I-H	*IDUA*	*-Idua*-W402X mouse	W402X (UAG) mouse locus	-Amikacin -G418 -Gentamicin -* NB54 -* NB84 -Paramomycin * 30 mg/kg administered SQ once for 2 weeks	-MEF studies showed that NB84 and NB54, are more effective at suppressing W402X than traditional aminoglycosides tested (more enzyme; lower GAGs; improved lysosomal morphology) -2-week in vivo treatment with NB54 and NB84 showed while both drugs led to a significant GAG decrease in multiple tissues (spleen, heart, brain) -NB84 was more efficient. -While brain GAGs were reduced, brain GM2 and GM3 gangliosides were not.	Wang et al. [67]
2012	MPS VI	*ARSB*	-cultured patient fibroblasts	-R315X (UGA) -R327X (UGA) -Q456X (UAA) -Q503X (UAG)	-Gentamicin -PTC124	-No increase in enzyme activity observed with gentamicin treatment -Significant increase in enzyme activity observed with PTC124 treatment in all cells with the exception of the Q503X cell line -Lysosome size reduced with PTC124 treatment -ARSB protein undetectable by western blotting	Bartolomeo et al. [68]
2013	MPS I-H	*IDUA*	*Idua*-W402X mouse	W402X (UAG) mouse locus	-Gentamicin -NB84 NMD inhibitors: -Caffeine -NMDI-1 (30 mg/mL gentamicin administered SQ once daily for 14 days +/− 5 mg/mL NMDI-1 administered SQ once daily on days 12–14	-Combining NMD inhibitors NMD-1 or caffeine with either gentamicin or NB84 enhanced enzyme activity and GAG reduction in MEFs -NMDI-1 also enhanced the ability of gentamicin to restore enzyme activity, normalize lysosome enzyme proliferation, and reduce GAG accumulation in the brain and spleen -No ill effects were observed with NMDI-1 administration	Keeling et al. [69]
2014	MPS I-H	*IDUA*	*-Idua*-W402X mouse	W402X (UAG) mouse locus	-NB84 (30 mg/kg administered SQ twice weekly for 28 weeks)	-Significant increase in enzyme activity in multiple tissues (more activity obtained when treatment was initiated early) -Tissue GAG accumulation reduced -neuroinflammation reduced -Improved heart morphology & function -Improved bone morphology -Improved activity levels	Gunn et al. [70]
2015	Infantile neuronal ceroid lipofuscinosis	*PPT1 or CLN1*	*-Cln1*-R151X mouse	R151X (UGA) mouse locus	PTC124 (10 mg/kg administered 4 times daily for 2 days)	Significant increase in PPT1 activity in liver and muscle, but not in brain, heart, lung, or kidney	Thada et al. [71]
2015	^a^ MPS VI ^b^ MPS IIIB ^c^ MPS IIIC ^d^ Niemann-Pick A/B	*^a^ ARSB* *^b^ NAGLU* *^c^ HGSNAT* *^d^ SMPD1*	-Cultured patient fibroblasts -Cell-free translation system -cDNAs in COS7 cells	-^a^ W146X (UGA) -^a^ W322X (UGA) -^a^ Q503X(UAG) -^b^ W168X (UAG) -^b^ Q566X (UAG) -^c^ R203X (UGA) -^c^ R384X (UGA) -^c^ W403X (UGA) -^d^ W168X (UAG) -^d^ Y313X (UAA) -^d^ R441X (UGA)	-BZ6 -BZ16 -G418 -Gentamicin -PTC124 -RTC13 -RTC14	-1%-4% of WT enzyme levels measured in W322X fibroblasts treated with gentamicin & ARSB protein detected by immunofluorescence -Enzyme restoration was not observed in MPS IIIB and IIIC fibroblasts with any of the drugs, but an increase in mRNA abundance was detected -Cell free systems showed RT stimulation of the three *SMDP1* mutations with G418 and gentamicin, but not the other drugs -RT was also observed with the HGSNAT mutations -No RT was observed with the ARSB mutations -In transfected COS7 cells G418, gentamicin, and PTC124 induced RT of the W146X, W168X, and Y313X mutations.	Gomez-Grau et al. [72]
2016	MPS I-H	*IDUA*	-Cell-free translation system	Q70X (UAG)	-Gentamicin -NB74 -NB124 -NB156 -NB157	-New synthetic compounds NB156 and NB157 were compared to their parent compounds and gentamicin. -Both new compounds showed an improved ability to suppress the Q70X mutation compared to the parent compounds and gentamicin.	Sabbavarapu et al. [73]

* Indicates in vivo dosing; a = ARSB, b = NAGLU, c = HGSNAT, d = SMPD1.
